# Effect of Maximal Apnoea Easy-Going and Struggle Phases on Subarachnoid Width and Pial Artery Pulsation in Elite Breath-Hold Divers

**DOI:** 10.1371/journal.pone.0135429

**Published:** 2015-08-18

**Authors:** Pawel J. Winklewski, Otto Barak, Dennis Madden, Agnieszka Gruszecka, Marcin Gruszecki, Wojciech Guminski, Jacek Kot, Andrzej F. Frydrychowski, Ivan Drvis, Zeljko Dujic

**Affiliations:** 1 Institute of Human Physiology, Medical University of Gdansk, Gdansk, Poland; 2 Faculty of Medicine, University of Novi Sad, Novi Sad, Serbia; 3 Department of Integrative Physiology, University of Split School of Medicine, Split, Croatia; 4 Department of Radiology Informatics and Statistics, Medical University of Gdansk, Gdansk, Poland; 5 Department of Computer Communications, Faculty of Electronics, Telecommunications and Informatics, Gdansk University of Technology, Gdansk, Poland; 6 National Centre for Hyperbaric Medicine, Institute of Maritime and Tropical Medicine, Medical University of Gdansk, Gdynia, Poland; 7 University of Zagreb School of Kinesiology, Zagreb, Croatia; The University of Tokyo, JAPAN

## Abstract

**Purpose:**

The aim of the study was to assess changes in subarachnoid space width (sas-TQ), the marker of intracranial pressure (ICP), pial artery pulsation (cc-TQ) and cardiac contribution to blood pressure (BP), cerebral blood flow velocity (CBFV) and cc-TQ oscillations throughout the maximal breath hold in elite apnoea divers. Non-invasive assessment of sas-TQ and cc-TQ became possible due to recently developed method based on infrared radiation, called near-infrared transillumination/backscattering sounding (NIR-T/BSS).

**Methods:**

The experimental group consisted of seven breath-hold divers (six men). During testing, each participant performed a single maximal end-inspiratory breath hold. Apnoea consisted of the easy-going and struggle phases (characterised by involuntary breathing movements (IBMs)). Heart rate (HR) was determined using a standard ECG. BP was assessed using the photoplethysmography method. SaO_2_ was monitored continuously with pulse oximetry. A pneumatic chest belt was used to register thoracic and abdominal movements. Cerebral blood flow velocity (CBFV) was estimated by a 2-MHz transcranial Doppler ultrasonic probe. sas-TQ and cc-TQ were measured using NIR-T/BSS. Wavelet transform analysis was performed to assess cardiac contribution to BP, CBFV and cc-TQ oscillations.

**Results:**

Mean BP and CBFV increased compared to baseline at the end of the easy phase and were further augmented by IBMs. cc-TQ increased compared to baseline at the end of the easy phase and remained stable during the IBMs. HR did not change significantly throughout the apnoea, although a trend toward a decrease during the easy phase and recovery during the IBMs was visible. Amplitudes of BP, CBFV and cc-TQ were augmented. sas-TQ and SaO_2_ decreased at the easy phase of apnoea and further decreased during the IBMs.

**Conclusions:**

Apnoea increases intracranial pressure and pial artery pulsation. Pial artery pulsation seems to be stabilised by the IBMs. Cardiac contribution to BP, CBFV and cc-TQ oscillations does not change throughout the apnoea.

## Introduction

Maximal apnoea performed by elite breath-hold divers results in extreme hypercapnia and hypoxia. Resulting chemostress augments sympathetic excitation, which in turn increases the blood pressure (BP) contribution to cerebral blood flow (CBF) [1,2]. Furthermore, hypercapnia causes cerebral arterial vasodilation [3,4,5] and can alter the pulse wave transmission characteristics of the cerebral vasculature by altering their Windkessel properties [5]. In addition, progressive carbon dioxide retention results in increases of intracranial pressure (ICP) [6,7,8]. The increased ICP may, in turn, impair internal jugular venous outflow, and does not allow for dampening the pulsation energy and actually exaggerates the pulsatile flow [9,10].

A maximal apnoea performed by elite apnoea divers consists of two distinct phases. The first phase, the so-called easy-going phase, is a quiescent period where respiratory neuromuscular activity is voluntarily suppressed, the glottis is closed, and there are no movements of the chest. The beginning of the second period, the struggle phase, is marked by the onset of involuntary breathing movements (IBMs) that increase in both magnitude and frequency until the end of the apnoea [11,12,13]. Cross et al. [13] reported that increases in both BP and CBF velocity in the IBMs phase were primarily due to increasing cardiac output (CO). Furthermore, during the IBMs phase, an increase in CBF velocity variability at the IBMs passband frequencies (0.20–0.80 Hz) was proposed [13]. Contrary, Willie et al. [14] indicated that the IBMs per se do not augment CBF.

There is accumulating evidence that CO is involved in the regulation of CBF. Georgiadis et al. [15] was the first to demonstrate long-term brain microcirculation adaptation to decreased CO in patients with chronic left ventricle failure. Specifically, a significant relationship between the decline in the left ventricle ejection fraction (LVEF) and the reduction in cerebrovascular reactivity was reported [15]. Ogoh et al. [16] indicated that CBF velocity in the middle cerebral artery (MCA) response to a rapid decline in systemic BP was highly related to the unloading of arterial baroreceptors. In an animal model with stable blood pressure (BP), positive correlations between changes in pial artery pulsation and LVEF, and between the systolic–diastolic cerebral blood volume fraction and LVEF were described [17]. Li et al. [18] reported a negative correlation between cerebral oxygenation and CBF velocity at respiratory and cardiac frequencies. Finally, a decrease in cardiac contribution to pial artery pulsation and blood pressure oscillation at the end of apnoea in normal subjects has recently been postulated [19].

Non-invasive assessment of pial artery pulsation became possible due to a recently developed method based on infrared radiation (IR) called near-infrared transillumination/backscattering sounding (NIR-T/BSS). In contrast to near-infrared spectroscopy (NIRS), which relies on the absorption of infrared light (IR) by haemoglobin [18], NIR-T/BSS uses the subarachnoid space (SAS) filled with translucent cerebrospinal fluid as a propagation duct for IR [20]. Thus, NIR-T/BSS enables the assessment of instantaneous changes in SAS width in humans (sas-TQ). Fast oscillations in the width of the SAS, further referred to as the cardiac component of subarachnoid width pulsation (cc-TQ), results from heart-generated pial artery pulsation. NIR-T/BSS high sampling frequency (70 Hz) allows for signal analysis up to 5 Hz. The power spectrum density of cc-TQ shows clear peaks at the fundamental frequency (f_0_) and its harmonics (f_1_, f_2_, f_3_) [21]. Changes in the SAS width correlate with ICP to a considerable extent, providing sufficient evidence of changes in ICP by measurements of sas-TQ [22,23].

We hypothesised that maximal apnoea in elite breath-hold divers will decrease in SAS width, suggesting an ICP increase. It will also augment the pial artery pulsation amplitude, as previously described in normal volunteers [8]. Furthermore, we expected no change in the cardiac contribution to pial artery pulsation, BP and CBF velocity oscillations throughout the apnoea, as measured by wavelet coherence between the analysed signals, due to better-developed protective mechanisms in elite apnoea divers than in normal volunteers.

## Materials and Methods

### Subjects

The experimental group consisted of seven breath-hold divers (six men and one woman). At the time of the study, they were all apparently healthy. The demographic data of the study subjects are shown in Table 1.

**Table 1:** Characteristic of the study participants.

Breath-hold divers	1	2	3	4	5	6	7
Height	168	193	188	189	192	188	188
Weight	53	100	79	82	91	80	94
Age	31	34	31	19	19	27	25
Diving experience (years)	8	7	8	3	2	5	7
Personal best (minutes)	5'40''	5'30''	7'10''	4'01''	3'32''	7'43''	4'40''

### Experimental design

**Fig 1:**
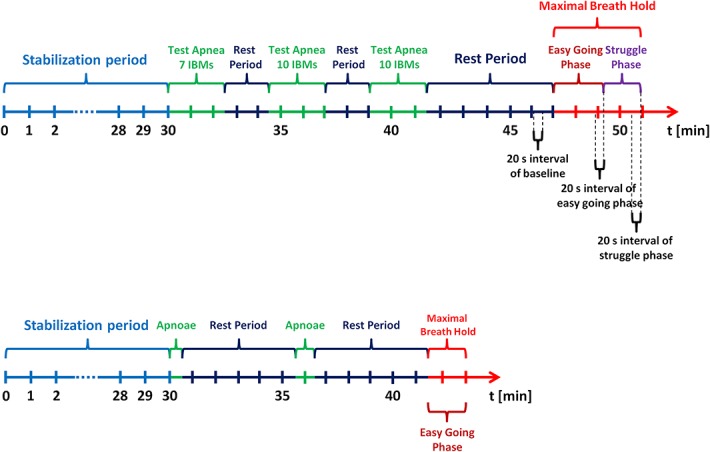
Schematic representation of study design in breath-hold divers (upper tracing) and normal healthy volunteers (lower tracing).

All experimental procedures were performed in accordance with the Declaration of Helsinki on the treatment of human subjects and the Ethical Committee of the University of Split School of Medicine approved this study. Informed written consent was obtained from each subject. All experiments were carried out in a climatised room in the morning hours. The participants arrived at the laboratory 30–45 min before the start of the experiments for instrumentation and an explanation of the procedures. They abstained from caffeine for at least 12 h and from food for at least 4 h prior to the test. In each subject, dynamic spirometry (Quark PFT, Cosmed, Rome, Italy) was evaluated in the upright posture. Measurement of height and weight was performed. After emptying their bladders, the subjects rested in the supine position for 30 min to ensure stabilisation of the cardiovascular parameters. Before maximal breath-hold apnoea, the subjects went through preparation procedure consisting of: apnoea until seven IBMs, two minutes rest, apnoea until 10 IBMs, two minutes rest, apnoea until 10 IBMs followed by five minutes 30 seconds rest. Then, during testing, each participant performed one maximal breath-hold. The subjects were instructed not to hyperventilate before the apnoea. Graphical presentation of the study design is shown in Fig 1 (upper tracing).

The results obtained from breath-hold divers were compared to data coming from previously described normal subjects [8]. Briefly, the group consisted of 20 healthy, nonsmoking volunteers: nine men (age 26.3±8.4) and eleven women (age 20.1±2.0). After baseline measurements, volunteers performed consecutive apnoeas lasting 30, 60 s followed by maximal breath-hold. Apnoeas were separated by 5 min rest intervals. The subjects were instructed not to hyperventilate before the apnoea. Graphical presentation of the study design is shown in Fig 1 (lower tracing) [8]. The results from maximal breath-hold were comparison with apnoe in breath-hold divers.

Heart rate (HR) was determined using a standard ECG. BP was assessed using a pneumatic cuff placed around the middle finger of the nondominant hand (Finometer, Finapress Medical Systems, Arnhem, the Netherlands). Finger BP was calibrated against the brachial BP. SaO_2_ was continuously monitored using pulse oximetry (Poet II, Criticare Systems, Waukesha, WI), with the probe placed on the middle finger of the dominant hand. A pneumatic chest belt was used to register thoracic and abdominal movements. Cerebral blood velocities of the right and left MCA were measured using a 2-MHz pulsed transcranial Doppler ultrasound system (ST3, Spencer Technologies, Seattle, WA, US). A specialised headband fixation device (model M600 bilateral head frame, Spencer Technologies) was used to secure the probes in position. Left ventricular stroke volume (SV) was computed from the arterial pulse wave using the improved Modelflow algorithm [24]. Cardiac output (CO) was taken as the product of HR and SV. Expired air was sampled from the mouthpiece and the end-tidal CO_2_ (EtCO2) was measured by a gas analyser (Poet II, Criticare Systems, Waukesha, WI, US). Changes in the amplitude of the pial artery pulsation and in the width of SAS with NIR-T/BSS were recorded using the head-mounted sensors of the SAS 100 Monitor (NIRT sp. z o.o., Wierzbice, Poland). The theoretical and practical foundations of the NIR-T/BSS method have been published before [20,21]. All variables were continuously recorded or videotaped and the signals were digitally recorded for further analyses.

### Wavelet analysis

**Fig 2:**
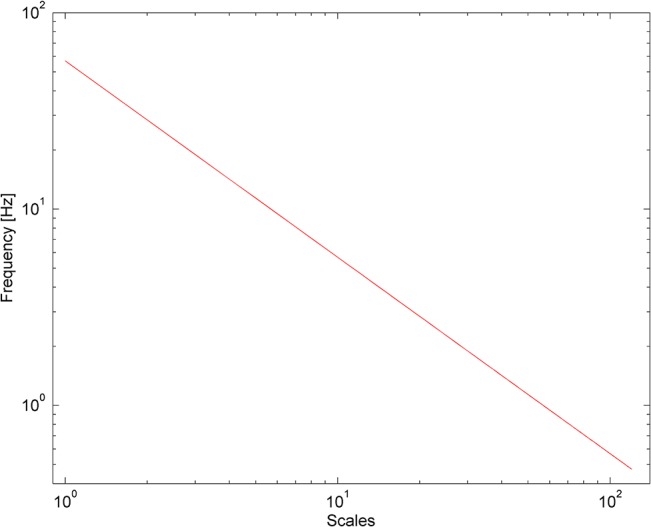
Transition from scales used in wavelet transform analysis to frequency values.

Wavelet transform is a method that transforms a time signal from the time domain to the time-frequency domain. The definition of the wavelet transform is:

$$W\left(s,t\right)=\frac{1}{\sqrt{s}}\int_{-\infty }^{+\infty }\varphi \left(\frac{u-t}{s}\right)g\left(u\right)du,$$ (1)

where *W*(*s*, *t*) is the wavelet coefficient, *g*(*u*) is the time series and *φ* is the Morlet mother wavelet, scaled by factor *s* and translated in time by *t*. The Morlet mother wavelet is defined by the equation:

$$\varphi \left(u\right)=\;\frac{1}{\sqrt[{4}]{\pi }}e^{-i2\pi u}e^{-0.5u^{2}},$$ (2)

where $=\sqrt{-1}$. The reason for using the Morlet wavelet is its good localisation of events in time and frequency due to its Gaussian shape [19,25]. The frequency is inversely proportional to its corresponding scaling factor *s* (see Fig 2 in log–log scale). The wavelet transform was calculated in the frequency interval from 0.47 to 56.7 Hz.

Wavelet coherence (WCO) and wavelet phase coherence (WPCO) were estimated using the Matlab function ‘wcoher.m’ with ‘morl’ (Morelet function) as a mother wavelet and a scale from 1 to 120, corresponding to a frequency interval of 0.47 to 56.7 Hz. A detailed description with clear examples can be found at: http://www.mathworks.com/help/wavelet/examples/wavelet-coherence.html. In all calculations, we estimated a normalised value of WCO:

$${\text{w}}_{12}=\frac{{\text{w}}_{1}{\left(\text{s},\text{t}\right)}^{*}{\text{w}}_{2}\left(\text{s},\text{t}\right)}{\sqrt{{\text{w}}_{1}{\left(\text{s},\text{t}\right)}^{2}}\sqrt{{\text{w}}_{2}{\left(\text{s},\text{t}\right)}^{2}}}$$ (3)

where w_1_(s,t) (w_2_(s,t)) is the wavelet coefficient for the first (the second) signal and ^*^ indicates a complex conjugate. We observed stronger coherence when the WCO value increased. The value of WPCO is between 0 and 1. When two oscillations are unrelated, their phase difference continuously changes with time, thus their WPCO approaches zero. If the WPCO is around 1, the two oscillations are related and significant coherence is observed.

### Statistical analysis

Two particular periods of apnoea were examined: the last 20 s of the easy phase and the last 20 s at the end of apnoea. Since no significant differences were noted between the left and right hemispheres with respect to sas-TQ, cc-TQ, CBFV, WCO and WPCO, these variables from the left and right sides were combined for statistical analysis.

A Wilcoxon signed-rank test was used to compare changes in WCO, WPCO, sas-TQ, cc-TQ, systolic BP (SBP), diastolic BP (DBP), HR, SV, CO, CBFV and SaO_2_ in response to apnoea.

To compare results between healthy volunteers and breath-hold divers we used a non-parametric Mann Whitney U test. We compared changes between in cc-TQ, sas-TQ, HR, SBP, DBP, CBFV, SaO_2_ and CO_2_.

## Results

The measurements obtained at the end of the easy phase and at the end of breath-holding are presented in Table 2 and Table 3.

**Table 2:** Effects of apnoea (225.66±72.61 s; mean ±SD) on cc-TQ, sas-TQ, HR, SBP, DBP, SV, CO, CBFV, and SaO_2_. Data presented as mean values and standard deviations (SD). All % changes are calculated with reference to baseline values.

	Baseline	End of easy phase	Change %	End of apnoea	Change %
Mean cc-TQ (AU)	52.8±7.1	82.3±17.3**	+55.3	82.8±16.4**	+55.3
Amplitude of cc-TQ (AU)	56.9±7.4	119.3±26.1**	+109.7	123.7±28.9**	+117.4%
sas-TQ (AU)	584.4±281.3	467.4±186.9***	-20.02	429.9±160.9***	-26.4
HR (beats*sec^-1^)	63.1±15.9	59.5±12.4^NS^	-5.56	64.8±15.9^NS^	+2.91
SBP (mmHg)	121.3±9.2	145.5±13.2*	+45.5	173.1±31.7**	+42.5
DBP (mmHg)	65.5±10.1	85.5±15.8*	+30.5	91.7±22.7*	+39.9
Amplitude of BP (mmHg)	77.3±10.7	93.7±22.9**	+21.2	105.1±27.4*	+36.0
SV	114.5±20.6	100.7±24.7^NS^	-12.1	103.7±37.7^NS^	-9.4
CO	6650±4298	6080±1977^NS^	-8.62	6998±3183^NS^	+5.2
Mean CBFV	50.1±18.6	81.5±24.7**	+62.7	99.3±26.1***	+98.3
Amplitude of CBFV	50.13±18.4	62.2±31.7**	+24.1	67.9±35.1**	+35.4
SaO_2_	99±0.0	95.8±2.9**	-3.2	83.7±14.7**	-15.5

*P < 0.05

**P < 0.01

***P < 0.001; NS not statistically significant; sas-TQ–the subarachnoid component of the transillumination quotient (the subarachnoid width); cc-TQ–cardiac component of transillumination quotient (pial artery pulsation); BP–blood pressure; SBP–systolic BP; DBP–diastolic BP; HR–heart rate; SV–stroke volume; CO–cardiac output; CBFV–cerebral blood flow velocity; SaO2—oxyhemoglobin saturation; AU–arbitrary units; mm Hg—millimeters of mercury; s–seconds

**Table 3:** cc-TQ, sas-TQ, HR, SBP, DBP, SV, CO, CBFV, and SaO_2_ percent change between end of easy phase and end of apnoea. Data presented as mean values and standard deviations (SD). All % changes are calculated with reference to baseline values.

	End of easy phase	End of apnoea	Change %
Mean cc-TQ (AU)	82.3±17.3	82.8±16.4^NS^	0
sas-TQ (AU)	467.4±186.9	429.9±160.9^*^	-8.7
HR (beats^*^sec^-1^)	59.5±12.4	64.8±15.9^NS^	+8.2
SBP (mmHg)	145.5±13.2	173.1±31.7^*^	+15.9
DBP (mmHg)	85.5±15.8	91.7±22.7^*^	+6.7
SV	100.7±24.7	103.7±37.7^NS^	+2.9
CO	6080±1977	6998±3183^NS^	+13.1
Mean CBFV	81.5±24.7	99.3±26.1^*^	+17.9
SaO_2_	95.8±2.9	83.7±14.7^*^	-14.5

*P < 0.05; NS not statistically significant; sas-TQ–the subarachnoid component of the transillumination quotient (the subarachnoid width); cc-TQ–cardiac component of transillumination quotient (pial artery pulsation); BP–blood pressure; SBP–systolic BP; DBP–diastolic BP; HR–heart rate; SV–stroke volume; CO–cardiac output; CBFV–cerebral blood flow velocity; SaO2—oxyhemoglobin saturation; AU–arbitrary units; mm Hg—millimeters of mercury; s–seconds

The mean SBP, DBP and CBFV increased compared to baseline at the end of the easy phase and were further augmented during the struggle phase by IBMs. cc-TQ increased compared to baseline at the end of the easy phase and remained stable during the IBMs. HR, SV and CO did not change significantly throughout the apnoea, although a trend towards decreasing during the easy phase and recovery during the IBMs was visible. Amplitudes of BP, CBFV and cc-TQ were augmented. sas-TQ and SaO_2_ decreased at the easy phase of apnoea and further decreased during the IBMs. The average apnoea duration was 225.7±72.6 s. EtCO_2_ increased from 39.7±1.0 mmHg (measured at baseline) to 48.0±1.5 mmHg (measured during first expiration) which was a 20.9% net difference (P<0.001).

Comparison between the results obtained from breath-hold divers and data coming from previously described normal subjects [8] is presented in Table 4.

**Table 4:** Effects of apnoea in healthy volunteers (91.1±23.1 s) and breath-hold divers (225.7±72.6 s) on cc-TQ, sas-TQ, HR, SBP, DBP, CBFV, and SaO_2_. Data presented as mean values and standard deviations (SD). All % changes are calculated with reference to baseline values.

	End of apnoea (healthy volunteers) Change %	End of apnoea (breath-hold divers) Change %	Statistical significance
Mean cc-TQ (AU)	+46,7	+55.3	NS
sas-TQ (AU)	-26,7	-26.4	NS
HR (beats*sec^-1^)	-3.2	+2.91	NS
SBP (mmHg)	+29.2	+42.5	P < 0.05
DBP (mmHg)	+24.1	+39.9	P < 0.05
Mean CBFV	+73.3	+98.3	P < 0.05
SaO_2_	-1.1	-15.5	P< 0.01
EtCO_2_	+19.9%	+20.9%	NS

NS not statistically significant; sas-TQ–the subarachnoid component of the transillumination quotient (the subarachnoid width); cc-TQ–cardiac component of transillumination quotient (pial artery pulsation); BP–blood pressure; SBP–systolic BP; DBP–diastolic BP; HR–heart rate; CBFV–cerebral blood flow velocity; SaO2—oxyhemoglobin saturation; AU–arbitrary units; mm Hg—millimeters of mercury; s–seconds

**Fig 3:**
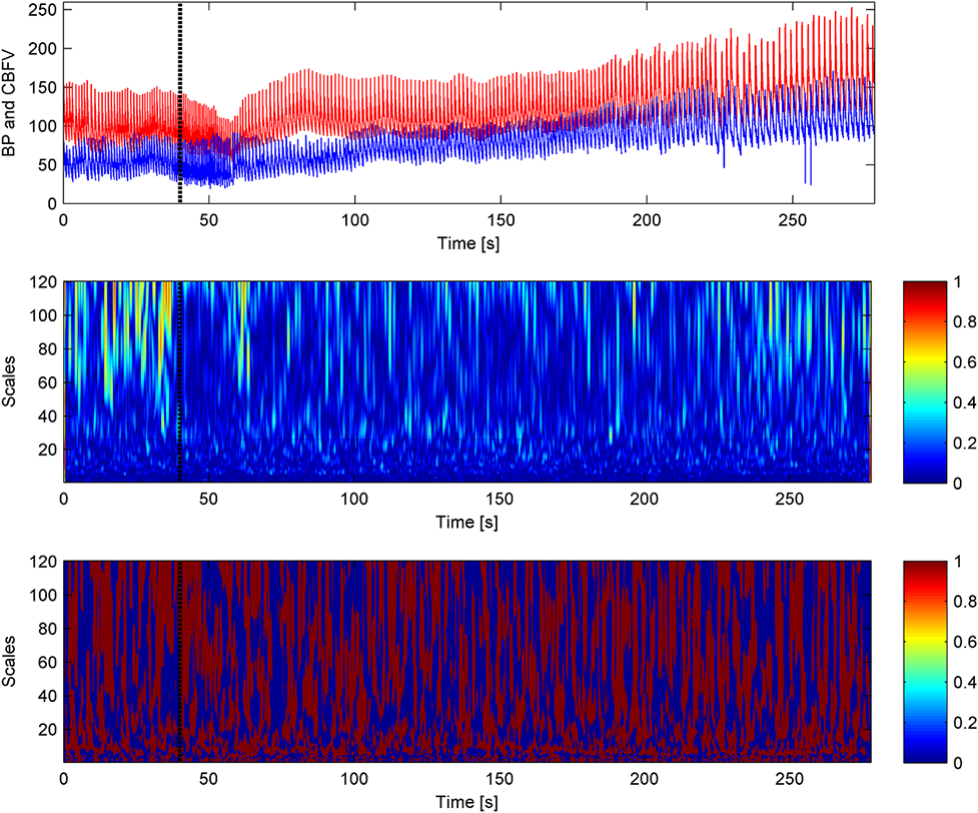
The black marker indicates the start of apnoea. BP (red) and CBFV (blue) signals are provided in the upper panel. WCO (middle panel) and WPCO (lower panel) remains stable throughout apnoea. Cardiac peak values at 40–60 scales (~1 Hz) are visible.

**Fig 4:**
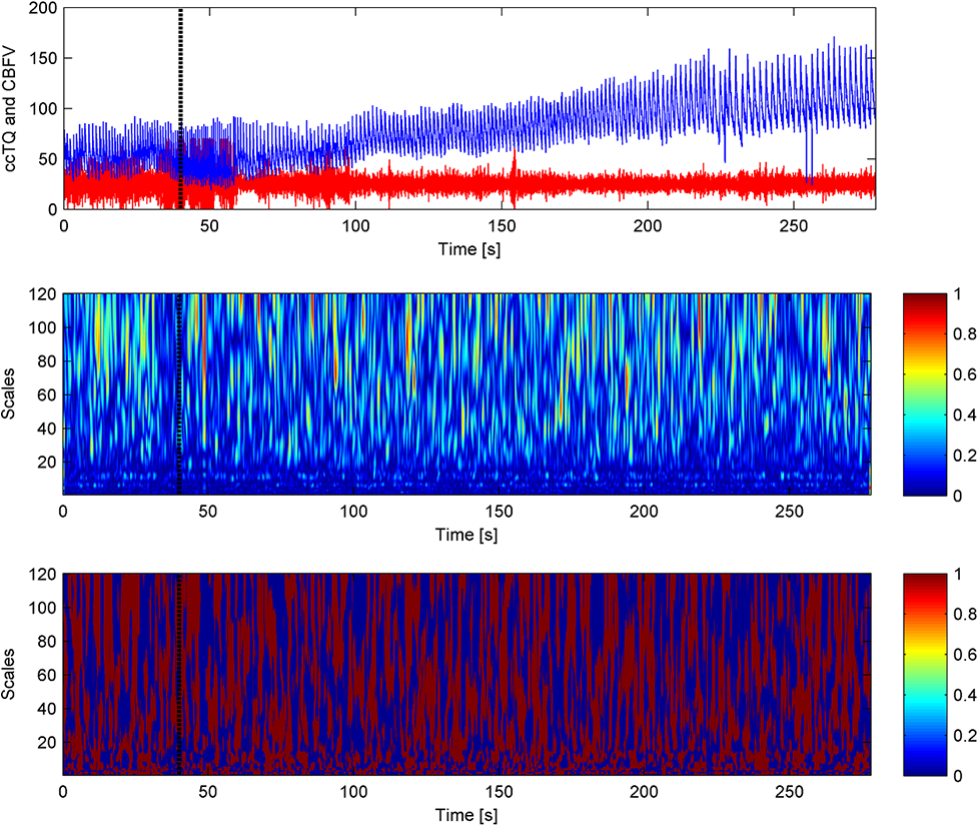
The black marker indicates the start of apnoea. CBFV (blue) and cc-TQ (red) signals are provided in the upper panel. WCO (middle panel) and WPCO (lower panel) remains stable throughout apnoea. Cardiac peak values at 40–60 scales (~1 Hz) are visible.

**Fig 5:**
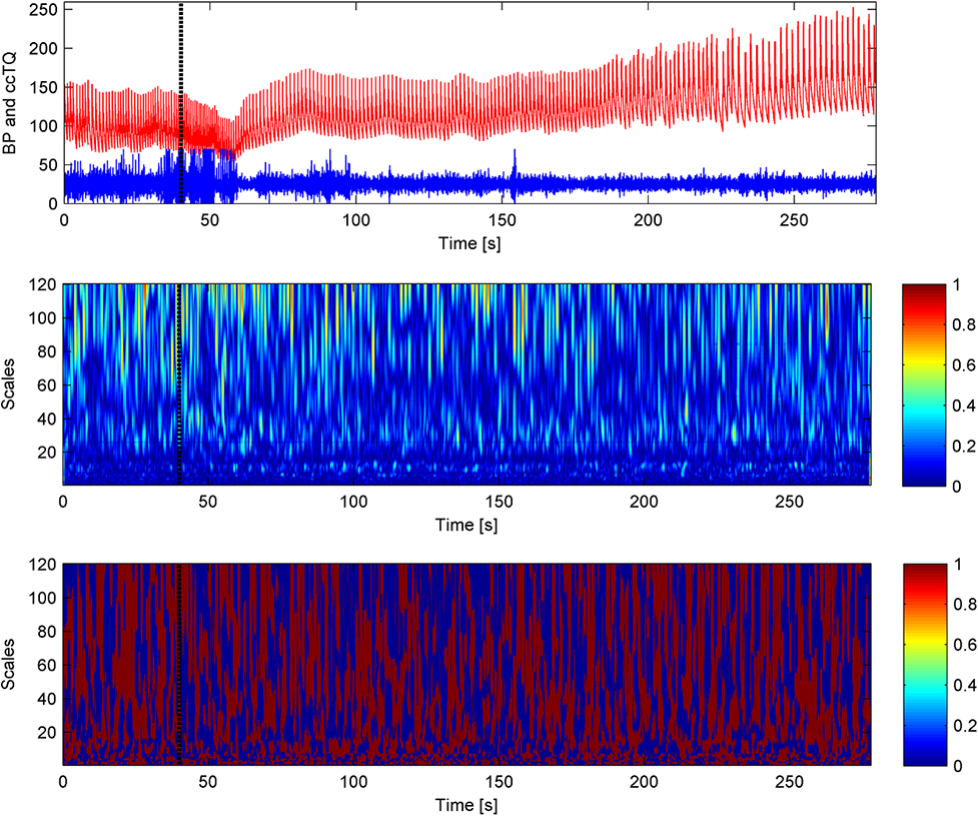
The black marker indicates the start of apnoea. BP (blue) and cc-TQ (red) signals are provided in the upper panel. WCO (middle panel) and WPCO (lower panel) remains stable throughout apnoea. Cardiac peak values at 40–60 scales (~1 Hz) are visible.

The typical time courses of BP, CBFV, and cc-TQ signals, as well as their respective WCOs and WPCOs are shown in Figs 3, 4 and 5. There were no changes in the cardiac contribution to oscillations between BP and CBFV signals, BP and cc-TQ signals and CBFV and cc-TQ signals throughout the breath-hold (Table 5).

**Table 5:** Wavelet coherence and phase coherence between BP, CBFV and cc-TQ during baseline, end of easy phase and the last 20 s of apnea (normalized values) at cardiac frequency.

BP vs. ccTQ	Baseline(mean ± sd)	End of easy phase (mean ± sd)	p–End of easy phase vs Baseline	End of apnoea (mean ± sd)	p–End of apnoea vs Baseline
Wavelet Coherence	0.14±0.04	0.15±0.05	NS	0.16±0.05	NS
Wavelet Phase Coherence	0.73±0.1	0.8±0.13	NS	0.65±0.14	NS
BP vs. CBFV	Baseline (mean ± sd)	End of easy phase (mean ± sd)	p–End of easy phase vs Baseline	End of apnoea (mean ± sd)	p–End of apnoea vs Baseline
Wavelet Coherence	0.2±0.03	0.18±0.03	NS	0.19±0.05	NS
Wavelet Phase Coherence	0.69±0.18	0.78±0.15	NS	0.71±0.12	NS
CBFV vs. ccTQ	Baseline (mean ± sd)	End of easy phase (mean ± sd)	p–End of easy phase vs Baseline	End of apnoea (mean ± sd)	p–End of apnoea vs Baseline
Wavelet Coherence	0.17±0.04	0.16±0.03	NS	0.15±0.07	NS
Wavelet Phase Coherence	0.7±0.14	0.73±0.07	NS	0.69±0.15	NS

^NS^ Not statistically significant; cc-TQ–cardiac component of transillumination quotient (pial artery pulsation); BP–blood pressure; CBFV–cerebral blood flow velocity; sd–standard deviation

## Discussion

There are three main findings of this study: 1) apnoea decreases SAS width, which clearly indicates ICP elevation, 2) pial artery pulsation augments during the easy-going phase and seems to be stabilised by the IBMs, and 3) there is no change in cardiac contribution to BP, CBFV and pial artery pulsation oscillations throughout the apnoea.

Increases in ICP, as measured by a sas-TQ decline, and increases in pial artery pulsation, as assessed by the cc-TQ parameter, throughout the apnoea were recently described in normal subjects [8,19]. The investigated population in the above-mentioned studies consisted of young and healthy, but not highly trained, individuals and over 50% of the study population were females. Clearly, the apnoea in such a group of volunteers consisted of only the easy-going phase. The increase in ICP due to progressive CO_2_ retention has also been described in animals [6] and obstructive sleep apnoea subjects [7]. Hypercapnia is known to dilate pial artery and decrease pial arteriolar bed resistance [26,27]. In NIRT-BSS, it is seen as an increase in cc-TQ parameter [4,28]. Pial artery dilation leads to augmentation of CBF [27,29], and subsequently, to increases in cerebral blood volume [28]. Increases in cerebral blood volume augment ICP, as seen in NIRT-BSS as a decline in the sas-TQ [4,28].

Interestingly, cc-TQ increases were only seen during the easy phase of prolonged apnoea. During the IBMs, cc-TQ stabilised, although at a higher level. There are two possible explanations for this phenomenon. Both changes in ICP and cerebrospinal volume may directly affect the pial artery [22,23]. We can speculate that at some point, extensive pial artery dilation due to augmented ICP, CO_2_ concentration, flow and pressure could result in the loss of their elastic properties and thus stabilisation in the pulsatility. However, we did not observe any swings in cc-TQ pulsation synchronised with the IBMs as we had originally expected during passive transmission. The IBMs influence intrathoracic pressure [30]. Swings in intrathoracic pressure are most likely transmitted to the intracranial pressure via cerebrospinal fluid like in the Valsalva manoeuvre [23, 31]. Alternatively, the second part of prolonged apnoea is associated with enormous increases in sympathetic activity [32]. It has been postulated that the sympathetic nervous system may protect cerebral circulation from transient surges in BP [33,34,35,36]. Stabilisation of pial artery pulsation was seen in such circumstances as active neurogenic autoregulatory processes.

Quite surprisingly, the percentages of sas-TQ and cc-TQ changes by the end of apnoea in the previous studies in normal subjects [8,19] and in the current study were almost identical, despite differences in BP, CBFV and SaO_2_. It should be noted that measurements with the use of IR light (NIRS and NIR-T/BSS) do not allow for direct comparisons between subjects due to differences in skull bone parameters [20,37]. However, NIR-T/BSS, like NIRS, allows for direct within-subject comparisons [20,37] and as long as relative changes from baseline values are analysed, high between-subject reproducibility is observed [20,23]. Therefore, we compared the percent changes and it seems that the protective mechanisms in elite apnoea divers are much better developed than in normal subjects as similar changes in cerebral haemodynamic occur in apnoea divers only after two and half times the duration of apnoea compared to normal subjects. To further explore this assumption, we analysed the cardiac contribution to BP CBFV, BP cc-TQ and CBFV cc-TQ oscillations.

During apnoea, the course of cerebral haemodynamic is highly dynamic. To trace these responses properly, we applied an average of 20 s as a compromise between stable averages and sufficient time resolution. We decided to examine two particular periods of the apnoea: the last 20 s of the easy-going phase and the last 20 s of the end of the apnoea. These periods were compared to the 20 s at baseline (Fig 1). We used wavelet transform analysis, as it provides windows of adjustable lengths, thereby providing the benefit of showing high resolution at cardiac frequency. Compared with autoregressive estimation, wavelet transform is calculated directly from data, and the limitations of linear modelling and the choice of model order are thus avoided [38]. We, as well as others, have already used this method [18,19,25,38,39,40].

In normal subjects, apnoea decreases cardiac contribution to BP pial artery pulsation oscillations [19]. Declined coherence between BP and cc-TQ signals at cardiac frequency is likely due to increased vagal outflow to the heart during apnoea and/or increased Wendkessel properties of cerebral vasculature [19]. In this study, for the first time, we measured the chain of events, starting from systemic BP, through CBFV in MCA to pial artery pulsation. Quite surprisingly, we did not find any change in cardiac contribution to any of the analysed pairs of signals in apnoea divers. Neither the easy phase nor the IBMs affected the coherence between BP and CBFV, BP and cc-TQ and CBFV and cc-TQ at cardiac frequency. High sympathetic drive in apnoea divers probably stabilises cardiac contribution throughout the apnoea [14]. Stabilisation of cardiac contribution to pressure, flow and pial artery pulsation oscillations strengthen the notion that apnoea divers develop effective mechanisms of protecting their brains against hypoxia.

Jennum & Børgesen showed over twenty years ago that there is a strong correlation between the duration of apnoea and BP and elevated ICP, and between BP variations and elevated ICP in obstructive sleep apnoea (OSA) patients [41]. However, since that time, ICP has not received significant consideration with respect to the pathophysiology and management of OSA. Our study provides an additional mechanistic explanation for the results of Jennum & Børgesen. However, it should be taken into account that the study was performed in healthy breath-hold divers during wakefulness and in the absence of respiratory efforts. Therefore, the study does not mimic OSA, and the study results cannot be extrapolated directly to the clinical situation of OSA. Furthermore, respiratory efforts are known to modulate venous return to the heart and may directly affect baroreceptor output [42]. Nevertheless, the current study on breath-hold divers, and our previous study on normal healthy volunteers [8] establish reference for future studies on OSA subjects which are warranted.

The study has some limitations that need to be recognized. The number of subjects is relatively low. However, elite breath-hold divers represent a unique and limited population that stretch current physiological limits to the most extreme. For example muscle sympathetic nerve activity (MSNA), an index of efferent sympathetic nerve activity, was increased 20 fold at the end of maximal inspiratory apnea compared to baseline values (32). Therefore most of the studies are performed on a limited numbers of participants. Furthermore, results for each individual elite breath hold diver was presented as relative values compared to his/her baseline value.

To conclude, we have shown that apnoea in breath-hold divers is associated with SAS width decline, indicating increases in ICP. The pace of ICP rise seems to be slower than in normal subjects. Pial artery pulsation augments during the easy-going phase due to elevated CO_2_ concentration, ICP, BP and CBFV but then stabilises during the IBMs phase. There is no change in the cardiac contribution to BP, CBFV and pial artery pulsation oscillations throughout the apnoea. Taken together, our results suggest that apnoea divers develop mechanisms protecting their brains against hypoxia and hazardous hemodynamic environments.
